# EXAMINING SITUATION SELECTION ACROSS THE LIFESPAN USING A NOVEL DAY RECOLLECTION METHOD

**DOI:** 10.1093/geroni/igae098.4174

**Published:** 2024-12-31

**Authors:** Judy Kwak, Patrick Hill

**Affiliations:** Washington University in St. Louis, St. Louis, Missouri, United States; Washington University in St. Louis, St. Louis, Missouri, United States

## Abstract

Situation selection is the process by which people approach or avoid certain people, places, or objects to regulate their emotions. Prior research suggests that older adults engage in situation selection more frequently than younger adults because they become more adept at avoiding situations that provoke negative emotions. However, research on these behaviors is limited, in part due to the lack of a reliable scale. The current study addressed this gap by surveying 304 adults ranging 18-79 years old (M=46, SD=16) from Prolific to test a novel measure of day recollection situation selection. First, participants reflected upon the previous day’s events and wrote about how they felt, as if writing in a diary. Next, their diary entry was presented in a read-only format at the top of the following page, where they answered questions about how they chose to approach or avoid situations. Exploratory factor analyses revealed a 3-factor structure reflecting distinct forms of situation selection: (1) approaching positive, (2) approaching negative, and (3) avoiding negative. Internal consistency of these subscales was high (alphas = 0.81–0.89), and intercorrelations were small-to-moderate (rs = -0.04–0.35). No age differences were found in approaching positive or avoiding negative situations (rs = -0.02– -0.07). However, older adults reported approaching negative situations less frequently than younger adults (r = -0.14). These findings suggest that older adults strategically select environments to enhance their well-being. With age, adults may prioritize emotional goals by selecting situations conducive to minimizing stressors while increasing positive experiences.

